# Hemoglobin targets for the anemia in patients with dialysis-dependent chronic kidney disease: a meta-analysis of randomized, controlled trials

**DOI:** 10.1080/0886022X.2018.1532909

**Published:** 2018-12-03

**Authors:** Yuqiu Ye, Hongyong Liu, Yanbing Chen, Yunqiang Zhang, Shaomin Li, Wentao Hu, Rongqian Yang, Zhesi Zhang, Linsheng Lv, Xun Liu

**Affiliations:** a Department of Nephrology, The Third Affiliated Hospital of Sun Yat-sen University, Guangzhou, China; b Division of Nephrology, Yuedong Hospital, The Third Affiliated Hospital of Sun Yat-sen University, Meizhou, China; c Medical Genetic Center, Guangdong Women and Children Hospital, Guangzhou, China; d Department of Biomedical Engineering, South China University of Technology, Guangzhou, China; e Operation Room, The Third Affiliated Hospital of Sun Yat-sen University, Guangzhou, Guangdong, China

**Keywords:** Anemia, prognosis, dialysis, meta-analysis

## Abstract

**Background:**

Anemia is extremely common among dialysis patients and underlies some of the symptoms associated with reduced kidney function, including fatigue, depression, reduced exercise tolerance, and dyspnea.

**Objectives:**

A clearer cognition of the prognosistic impact of hemoglobin (Hb) or hematocrit (Hct) target for the outcomes of dialysis patients is urgent. This article aims to establish the suitable hemoglobin in order to provide clinical guidance.

**Methods:**

MEDLINE, EmBase, the Cochrane Library and other databases were searched with both MeSH terms and keywords to gather randomized controlled trials that assessed all-cause mortality, cardiovascular events, fistula thrombosis, infectious diseases and transfusion among dialysis-dependent patients using erythropoiesis-stimulating agents. The meta-analysis was accomplished via Revman 5.3 version.

**Findings:**

Totally, nine eligible studies were included, with study subjects involving 3228 patients. There was a significantly higher risk of fistula thrombosis without heterogeneity (RR 1.34, 95% CI 1.15–1.55; *p* < 0.05) in the higher Hb target group than in the lower Hb target group in the fixed effects model. However, no significant difference was found in all-cause mortality in the fixed effects model (RR 1.09, 95% CI 0.93–1.27; *p* = 0.30), cardiovascular events (RR 0.77, 95% CI 0.31–1.92; *p* = 0.58), infectious diseases (RR 0.69, 95% CI 0.24–1.96; *p* = 0.49) and transfusion (RR 0.92, 95% CI 0.42–1.99; *p* = 0.82) in the random effects model between the higher Hb target group and the lower Hb target group.

**Discussion:**

The results favor lower Hb target. To target lower Hb target when treating dialysis patients with anemia may decrease the risk of fistula thrombosis without increasing the risk of death, cardiovascular events, infectious diseases and transfusion.

## Introduction

In the United States, the prevalent population of patients with ESRD undergoing maintenance dialysis currently exceeds 460,000 [1]. It is global public health problem, because of its high prevalence, heavy economic burden and poor prognosis. Dialysis patients easily suffered from anemia due to endogenous erythropoietin deficiency, shortened RBC survival, and uremic inhibitors [2]. Recombinant human erythropoietin and the analogs are widely used to treat anemia. To our best knowledge, anemia aggravates exercise tolerance, cognitive competence and reduces quality of life in dialysis patients [3–5]. Anemia also can exert adverse influence on cardiovascular system such as left ventricular hypertrophy or dilation, arrhythmia, and myocardial ischemia [6,7]. Reversing anemia may reduce the risk. However, it is reported that adverse effects of the higher hemoglobin level include the development of systemic hypertension, site access thrombosis in dialysis patients with arteriovenous shunts and increased risk of cardiovascular events [8]. Interventional evidence has been pointing in a different direction. A meta-analysis [9] found that CKD patients including predialysis and dialysis with erythropoiesis-stimulating agents (ESAs) targeting the higher Hb do not lower mortality and may increase cardiovascular risk.

Kidney Disease Improving Global Outcomes (KDIGO) Guidelines have been developed for the anemia control targets, but there remains considerable controversy regarding the appropriate Hb or Hct levels as shown by the wide variation that still exists in anemia management practices between and within countries. The aim of this systematic review is to summarize the benefits and harms of lower versus higher Hb group in the treatment of the anemia of dialysis patients using existing randomized controlled trial data.

## Materials and methods

### Data sources and literature searches

We conducted a systematic review and meta-analysis according to the Preferred Reporting Items for Systematic Reviews and Meta-analyses (PRISMA) guidelines [10]. We electronically searched MEDLINE literature to collect all relevant literature using the search terms or synonyms ‘*Dialysis’, ‘hemodialysis’,* ‘*peritoneal dialysis’ hemoglobin, Hb, Hematocrit, Hct, ‘erythropoiesis stimulating agent, recombinant human erythropoietin, rhuEPO, darbepoetin, erythropoietin,’* from inception to 25th December 2017. Randomized controlled clinical trials were also identified via EMBASE (1974 to December 2017), the Cochrane Controlled Clinical Trials Register Database (through December 2017), the Cochrane Renal Group Specialized Register of Randomized Controlled Trials (through December 2017) and the ClinicalTrials.gov website.

**Figure 1.**
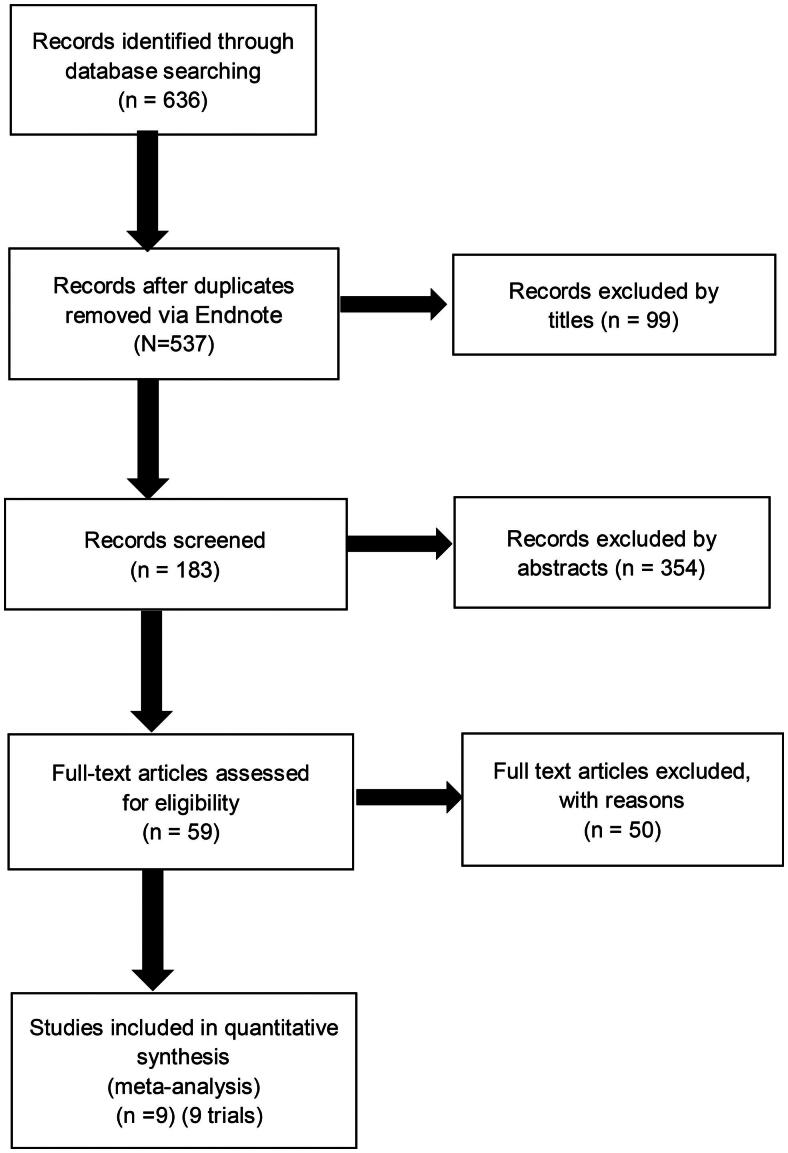
Flow diagram of studies considered for inclusion.

We also searched (manually) the abstracts of conference proceedings of the American Society of Nephrology from 1998 to 2017. However, we did not have access to RCTs that were not reported. Our searches have no restriction on language. Finally, we found 636 studies for the analysis. After screening, nine acceptable studies [11–19] were included in the analysis (Figure 1).

### Study selection

All RCTs that studied dialysis adults (age > 18 years) and compared higher Hb target to lower one was included. We excluded the studies as follows: duplicate publication; studies with inadequate facts; personal perspective, academic conferences, reviews and meta-analysis articles; animal and cell experiment; literature that studies predialysis patients; population age <18 years. Titles and abstracts were reviewed and evaluated by two reviewers independently, as well as the full-text articles.

### Data extraction and quality assessment

Data were independently extracted by two authors (Yuqiu Ye and Shaomin Li). Search strategies of all databases could be found in S1 Appendix 1. Finally, we found 636 studies for the analysis. After screening, nine studies were included in the analysis (Figure 1). The following data were extracted: country of origin; year of publication; sample size; mean age; percentage of men; mean or median follow-up time; the status of iron and ESAs usage; prevalence of diabetes; prevalence of hypertension; different Hb (or Hct) targets; number of endpoints outcomes.

We also extracted and mine data on trial characteristics, trial intervention. Regularly, several items such as independent randomization center, random allocation, blindness, adequate allocation concealment, intention-to-treat for RCTs were recorded. The quality of trials was assessed by Review Manager 5.3 (Oxford, UK) according to the Cochrane Handbook for Systematic Reviews of Interventions. Discrepancies concerning extraction and/or assessment of the quality of data were addressed by the third person if necessary.

### Synthesis and analysis of data

The software Review Manager 5.3 was used to implement meta-analysis. Risk ratios (RRs) were used to pool results for dichotomous outcomes (e.g. all-cause mortality). We use a fixed- (used if *I*^2^ < 25%) and a random-effects model (used if *I*^2^ > 50%) to analyze data. Ninety-five percent confidence intervals (95% CIs) were provided for all pooled estimates. Heterogeneity was assessed using the Cochrane Q test. *I*^2^ index (which describes the percentage of total variation across studies due to true heterogeneity rather than chance) and *p* values were also used. Publication bias was assessed using Funnel plots.

We performed a sensitivity analysis by removing the low-quality trials. We treated the trials with more than two ‘high risk’ according to Review Manager 5.3 as low-quality trials.

## Results

### Selection and characteristics of studies

A total of 636 potentially relevant citations were identified and screened. Eighty-six articles were retrieved for detailed evaluation, of which nine fulfilled the eligibility criteria (Figure 1). Detailed characteristics and a summary of all nine studies are displayed in Table 1. Multiple publications with no unique result were excluded from screened studies. However, unique results were extracted and studies (as well as abstracts) containing unique results were also displayed. The average age of each group ranged from 52.2 to 66.0 years. The sample size of the studies varied from 19 to 1233. Duration of follow-up was from 6 months to 48 months.

**Table 1.** Main characteristics of 27 studies selected for a meta-analysis.

References	Country	Population	Sample size	Age (I/C)	Male (%) (I/C)	Hypertension (%) (I/C)	Diabetes (%) (I/C)	Follow-up (month)
Bahlmann [12]	Germany	HD	NR	56/58	38.1/47.0	46/47	9/27	6
Besarab [11]	US	HD	12/12	65/64	50/52	71/69	54/58	29
Foley [13]	Canada	HD	NR	62/60	47/ 44,	NR	NR	12
Foley [14]	Canada/Europe	HD	NR	52.2/49.4	60.5/60.3	NR	NR	24
Furuland [15]	Europe	HD, PD	12/14	63/63	67/63	NR	19/20	19
Kuragano [16]	Japan	HD	11.5/18.2	60.2/53.4	33.3/29.4	NR	17.5/12.9	29
Parfrey [17]	Canada, Europe	HD	15.6/15.2	52.2/49.4	60/60	NR	NR	24
Roman [18]	US	HD	9/8	62/66	47.1/16.7	41.2/55.6	NR	13.5
Gaughan [19]	US	HD	16.7/15.4	53.7/45.3	77.8/40.0	NR	NR	6

**Table:** 

References	Intervention (I/C)	The doses of ESAs (U/kg/wk) (I/C)	The doses of iron (μg/dl) (I/C)	Ferritin values (ng/ml) (I/C)	Transferrin saturation values (%)	Higher Hb target (g/dl)(mean ± SD)	Lower Hbtarget(g/dl)(mean ± SD)	Higher Hb values actually reached (g/dl)(mean ± SD)	Lower Hb values actually reached (g/dl)(mean ± SD)
Bahlmann [12]	EPO /placebo	240/None	NR/NR	120/203	NR/NR	Hct 30–35	23	22–35%	23
Besarab [11]	EPO /placebo	146 ± 103/153 ± 119	NR/NR	391 ± 424/503 ± 442	26.8 ± 12.9/26.3 ± 12.0	Hct 42 ± 3	HCT 30 ± 3	42 ± 3	30 ± 3
Foley [13]	Epoetin/Epoetin	139/293	NR/NR	NR/NR	NR/NR	13.0–14.0	9.5–10.5	13.0–14.0	9.5–10.5
Foley [14]	Epoetin/Epoetin	7009U/wk/6183U/wk	NR/NR	NR/NR	NR/NR	13.5–14.5	9.5–11.5	13.5–14.5	9.5–11.5
Furuland [15]	Epoetin/Epoetin	150/NR	NR/NR	559 ± 421/487 ± 339	32 ± 16/31 ± 15	13.5–16.0	9.0–12.0	13.5–16.0	9.0–12.0
Kuragano [16]	EPO/EPO	4262 ± 481IU/wk /2390 ± 374 IU/wk	76.4 ± 33.1/78.9 ± 29.1	183.4 ± 40.7/212.3 ± 45.4	32.3 ± 2.3/29.3 ± 0.2	10.0–11.0	<10	10.5 ± 0.2	10.2 ± 0.2
Parfrey [17]	Epoetin/Epoetin	NR/NR	NR/NR	NR/NR	34.6 ± 14.91/34.2 ± 16.53	13.5–14.5	9.5–11.5	13.1 ± 0.9	10.8 ± 0.7
Roman [18]	EPO/EPO	37,600 ± 16,074 IU/wk/10,671 ± 7,236 IU/wk	NR/NR	NR/NR	NR/NR	14 ± 1	10 ± 1	12.7 ± 1.3	11.0 ± 0.8
Gaughan [19]	EPO/EPO + nandrolone	NR/NR	NR/NR	NR/NR	NR/NR	Hct 33.2 ± 4.5	28.3 ± 2.8	33.2 ± 4.5	28.3 ± 2.8

HCT: hematocrit; HD: hemodialysis; NR: not reported; PD: peritoneal dialysis.

### Effects of higher or lower Hb targets onall-cause mortality

**Figure 2.**
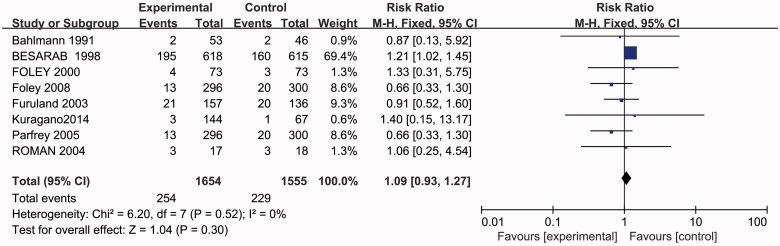
Metagraph of all-cause mortality.

In an analysis of eight studies with 3209 participants reporting on all-cause mortality, no significant difference was found in all-cause mortality in the fixed effects model (RR 1.09, 95% CI 0.93–1.27; *p* = 0.30) (Figure 2).

### Effects of higher or lower Hb targets on cardiovascular events

**Figure 3.**
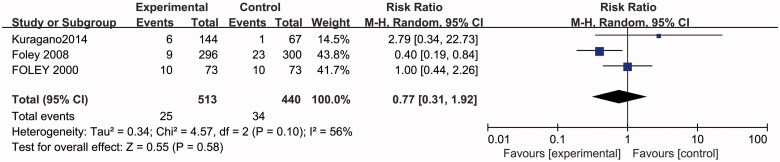
Metagraph of cardiovascular events.

Three studies with 953 participants reported on cardiovascular events. No significant difference was found in cardiovascular events in the random effects model with heterogeneity between studies (RR 0.77, 95% CI 0.31–1.92; *p* = 0.58) (Figure 3).

### Effects of higher or lower Hb targets on infectious diseases

**Figure 4.**
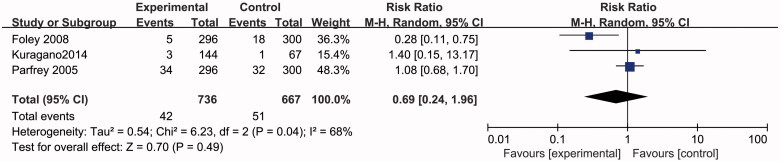
Metagraph of treatment of infectious diseases.

In an analysis of three studies with 1403 participants reporting on infectious diseases, no significant difference was found in infectious diseases in the random effects model (RR 0.69, 95% CI 0.24–1.96; *p* = 0.49) (Figure 4).

### Effects of higher or lower Hb targets on fistula thrombosis

**Figure 5.**
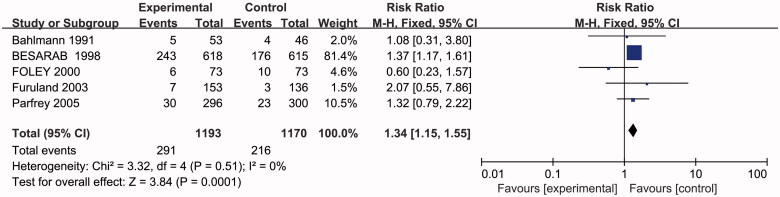
Metagraph of fistula thrombosis.

Five studies with 2363 participants reported on fistula thrombosis. A significant increase in the risk of fistula thrombosis was found in the higher Hb target group by 34% in the fixed effects model without heterogeneity between studies (RR 1.34, 95% CI 1.15–1.55; *p* < 0.05) (Figure 5).

### Effects of higher or lower Hb targets on transfusion

**Figure 6.**
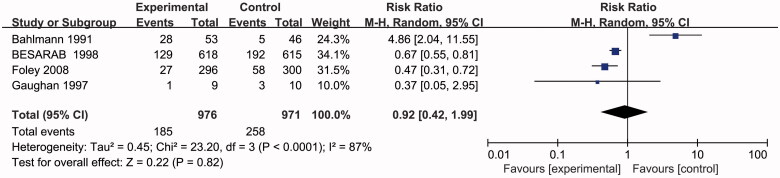
Metagraph of transfusion.

Four studies with 1947 participants reported on transfusion. No significant difference was found in transfusion between two groups in the random effects model (RR 0.92, 95% CI 0.42–1.99; *p* = 0.82) (Figure 6).

### Sensitivity analysis and publication bias

We conducted subgroup analyses and univariate meta regression (data not shown) to explore trial-level factors (patients, intervention, and design) that may affect the risks for transfusion. When the 4 primary trial designs were compared (placebo-controlled or low-dose ESA vs. high ESA comparator or plus nandrolone), the treatment comparison did not influence the risk for all clinical outcomes. When we explored the reasons for serious heterogeneity in the risk for transfusion, we found a higher risk in Gaughan 1997 with less participants, shorter follow-up and adjudication of outcomes was not blinded. We could not further explore the possible role of these factors on the risk for any outcome.

**Figure 7.**
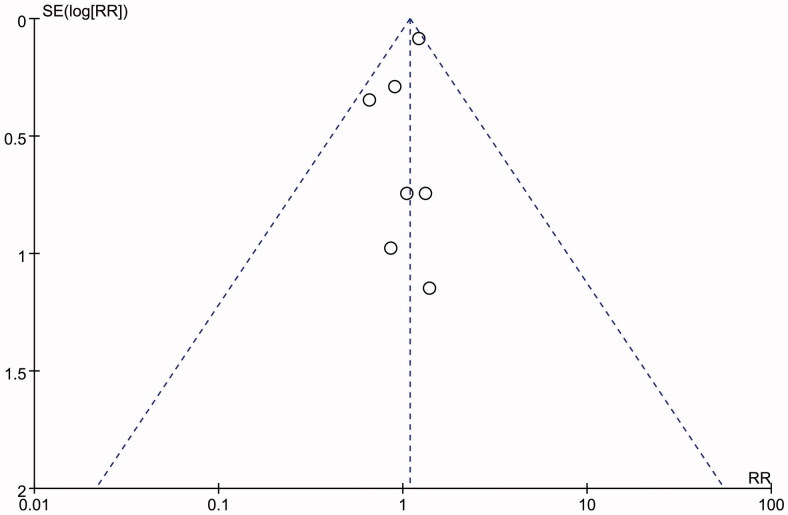
The funnel plot of this study.

Figure 7 showed a funnel plot of the studies included in this meta-analysis. All studies lie inside the 95% CIs, with an even distribution around the vertical, indicating no obvious publication bias.

## Discussion

To the best of our knowledge, this meta-analysis first evaluated current evidence from RCTs comparing different the Hb target groups in dialysis patients with anemia. The results suggested that compared with higher Hb target group, lower Hb target group significantly reduced the risk of fistula thrombosis. Meanwhile, no significant difference in all-cause mortality, cardiovascular events, infectious diseases and transfusion was observed between two groups.

The impact of maintaining different Hb targets on survival of dialysis patients was investigated previously in observational studies and RCTs, showing inconsistent results. The former suggested that lower hemoglobin (approximately 103g/L)was associated with death in hemodialysis patients [20]. Another observational study indicated that an Hb level ≥ 11 g/dL hemodialysis patients group was not associated with decreased mortality risk comparing reference Hb level of 10–11 g/dL after adjustment for multiple clinical variables [21]. However, recent meta-analyses of only RCTs with samples varying from 464 to 7902 participants indicated no reduction of all-cause mortality and even higher risk of death in CKD patients including dialysis and predialysis with higher Hb target (>13 g/dL) than those with lower ones [8,9,22]. In light of the deficit in the ability of observational studies to discover causality between intervention and clinical outcome, the present evidence seemed to suggest no favorable association between higher Hb level (>13 g/dL) and survival advantage in dialysis populations. However, the hemoglobin target is not clear. The best data among hemodialysis patients are from the Normal Hematocrit Trial (NHT), in which 1233 hemodialysis patients with cardiac disease, defined as heart failure or ischemic heart disease, and show that the group targeted to Hct 42 ± 3% (approximately Hb 14g/dl) had a higher risk of the combined endpoint of death or nonfatal myocardial infarction and the risk of thrombosis of grafts and fistulae comparing with Hct 30 ± 3% group (approximately Hb 10g/dl). We should not just to consider the Hb targets but also the Hb values actually reached in the two groups, the doses of ESAs and iron used in the two groups as well as the ferritin and transferrin saturation values. Most of study, the target is aligned with the Hb values actually reached. Most data of iron used in the two groups as well as the ferritin and transferrin saturation values were still missing.

Importantly, no significant difference was found in cardiovascular events between two groups, not in line with a recent meta-analysis investigating different doses of ESAs or to ESAs and to placebo in CKD patients including dialysis and predialysis [9]. The above meta-analysis showed that, in patients with CKD including dialysis and predialysis, treatment of anemia with ESAs targeting a higher hemoglobin value may increase cardiovascular risk. According to our result, lower hemoglobin target did not put dialysis patients at increased risk of cardiovascular events. However, trials included in our review reports did not give the cause of death. We tried to contact the authors but they did not reply. So, we can not specify whether cardiovascular events were fatal or not.

Our meta-analysis pooling five randomized controlled trials on treatment of anemia in dialysis patients showed that lower Hb targets significantly reduced the risk of fistula thrombosis by 34%. During a preliminary search, we identified a recently published meta-analysis on the same topic [9]. In accordance with the former conclusion, a higher hemoglobin target was associated with increased risk of vascular access thrombosis. Former meta-analysis did not include Furuland et al. [15], Bahlmann et al. [12] and Foley et al. [13], which were well designed randomized controlled trials. The result of our study with bigger sample is consistent with previous meta-analysis. The underlying mechanism that might partly be the facilitation of spontaneous platelet aggregation by EPO used to treat anemia and its interaction with blood coagulation factors, both leading to tendency of thrombosis [23,24].

Few meta-analyses reveal the association between HB target and infectious diseases. Infection is a common cause of death among hemodialysis patients. The iron supplement is commonly used to improve the hyporesponsiveness of ESAs treatment for dialysis patients. Former observational study investigated the risk factors and the outcome of bloodstream infections (BSIs) in hemodialysis patients [25]. The author made a conclusion that low hemoglobin (approximately 105 g/L) was significant risk factor. Recently, a prospective, observational, multicenter research show that patients with a stable target hemoglobin level had less risk for infectious diseases compared with other maintenance hemodialysis patients [26]. Some researcher formulated the hypothesis that a lower hemoglobin level may potentially be associated with risk of iron overload, which may lead to enhancement of bacterial growth and impairment of phagocytic function [27,28]. Previous studies have shown that iron overload, render hemodialysis patients more susceptible to bacteremia [27,28]. Our results show that no significant difference was found in transfusion for dialysis patients. However, we cannot identify the specific categories of infectious diseases and whether fatal or not.

A recent meta-analysis [29] shows the benefit of a higher HB target was the reduction of transfusion rate for predialysis and dialysis patients. The discretionary and abused use of blood products may have negative consequences including immune disorders, pulmonary complications, increased the chance of infection, longer intensive care unit stay, red blood cell (RBC) alloimmunization, and increased overall mortality [30–33]. In addition to the health risks associated with transfusions, blood products are an increasingly hospitalization expenses. It is reported that medical institutions in the United States pay approximately $225 USD per unit of RBCs, let alone but the triple cost of the administrative and labor costs associated with receipt, storage, transportation, and transfusion of the blood products [34]. As such, reducing the unnecessary use of blood products has the potential to control hospital costs associated with blood products and reduce transfusion-related morbidities. Higher Hb targets in dialysis and predialysis patients may reduce cost and prevent the health risks associated with transfusions. But we fail to conduct the risk benefit analysis or cost of ESA use. In detail, we cannot conduct subgroup analysis and assess number need to treat, differing risk populations, or whether trials of short duration in healthier patients can really assess the thrombotic or stroke risk of ESA's.

The KDIGO guideline suggested HB ≤ 11.5 g/dl for dialysis patients according to the upper HB boundary of the lower Hb level group in major ESA RCTs, and uncertainty still existed on the effect of Hb concentration between 11.5–13.0 g/dl [35]. In addition, emphasis was put on individual treatment balancing the pros and cons of ESA therapy and fistula thrombosis. In current study, the HB concentrations in two groups were approximately >130 g/L and 90–120 g/L. Based on current clinical data, a lower Hb target was recommended for dialysis patients. Importantly, a recent meta-analysis pooling dialysis and predialysis patients concluded that higher hemoglobin target was associated with increased risk of stroke [9]. In cases that tended to avoid higher hemoglobin-related complication such as hypertension thromboembolic diseases, in consideration of lower hemoglobin level not increase risk of death and cardiovascular disorders, maintaining a lower Hb concentration might be beneficial.

Chronic, low-grade inflammation is a common comorbid condition in CKD, and particularly in chronic dialysis patients. Inflammation also contributes to anemia and erythropoietin resistance [36], mediated by decreased erythropoietin production [37]. Human antigen R (HuR) is a member of the embryonic lethal abnormal vision (ELAV) family. As researches proceed, more and more proofs demonstrate its relationship with inflammation [38]. HuR is now suggested to play a pivotal role in glomerular nephropathy, tubular ischemia-reperfusion damage, renal fibrosis and even renal tumors. Future research will ultimately elucidate the therapeutic value of this novel target.

There is strong observational evidence that inflammation is high in chronic dialysis patients and that this is independently associated with numerous adverse clinical outcomes [39]. Targeting inflammation represents a potentially novel and attractive strategy. Inflammation induced renal tubular injury related interstitial fibrosis [40], and vascular endothelial dysfunction [41]. Endothelial-to-mesenchymal transition (EndMT) of glomerular vascular endothelial cells (GEnCs) is now considered to play a critical role in diabetic nephropathy (DN). NOD2 is newly discovered to be closely related to DN renal injury. A new article indicated that NOD2 has a regulatory role in EndMT via activation of MEK/ERK in high glucose-treated GEnCs [42]. New therapies targeting NOD2/MEK/ERK pathway is a promising strategy to treat DN endothelial dysfunction and reduce inflammation.

In this meta-analysis, there were several limitations that must be taken into consideration. First, we cannot specify the proper hemoglobin or hematocrit level in predialysis patients. In detail, over all studies the lower Hb boundary of the high Hb group overlapped the upper boundary of the low Hb group when compared among studies. Several studies included concentrated on the clinical outcome of different Hct levels and cannot explain the corresponding Hb levels. The missing element is the dose of ESA used in some studies. Second, the characteristics of studies; such as the sample, the follow-up period are hugely different, which might increase heterogeneity and bias the results. Thirdly, unpublished reports could not be identified, which might have biased our results. Finally, due to lack of relative articles of studying higher Hb (such as >150 g/L), we cannot summarize and give the answer of upper limit of Hb concentration.

## Conclusions

This meta-analysis pooling available RCTs suggested that targeting lower Hb when treating dialysis patients with anemia decrease risk of fistula thrombosis and had no significant effect on increased risk of death, cardiovascular events, infectious diseases and transfusion. In overall, our studies favor lower Hb target in dialysis patients.

## Supplementary Material

S1._Search_strategy.docx
