# Supplementary material for: Hemoglobin targets for the anemia in patients with dialysis-dependent chronic kidney disease: a meta-analysis of randomized, controlled trials
Source: Ren Fail. 2018 Dec 3;40(1):671–9. doi: 10.1080/0886022X.2018.1532909 (PMC6282462; doi:10.1080/0886022X.2018.1532909)
Supplement: S1._Search_strategy.docx [file IRNF_A_1532909_SM9989.docx]

Pubmed

(((((((((erythropoiesis stimulating agent[Text Word] OR recombinant human erythropoietin[Text Word]) OR rhuEPO[Text Word]) OR ESA[Text Word]) OR darbepoetin[Text Word]) OR epoetin[Text Word]) OR EPO[Text Word]) OR erythropoietin[Text Word]) AND (((Dialysis[Text Word] OR hemodialysis[Text Word]) OR haemodialysis[Text Word]) OR peritoneal dialysis[Text Word])) AND Randomized Controlled Trial[Filter]) OR (((((((((hemoglobin[Text Word] OR haemoglobin[Text Word]) OR Hematocrit[Text Word]) OR Haematocrit[Text Word]) OR Hb[Text Word]) OR Hbg[Text Word]) OR HCT[Text Word]) OR "hemoglobins"[MeSH Major Topic]) AND (((Dialysis[Text Word] OR hemodialysis[Text Word]) OR haemodialysis[Text Word]) OR peritoneal dialysis[Text Word])) AND Randomized Controlled Trial[Filter]) 636

**Embase**

'hemoglobin'/exp OR 'hematocrit'/exp OR hemoglobin OR haemoglobin OR hematocrit OR haematocrit AND ('hemodialysis'/exp OR 'peritoneal dialysis'/exp OR 'dialysis'/exp OR 'renal replacement therapy'/exp OR dialysis OR hemodialysis OR haemodialysis OR 'peritoneal dialysis' OR 'renal replacement therapy') OR ('hemodialysis'/exp OR 'peritoneal dialysis'/exp OR 'dialysis'/exp OR 'renal replacement therapy'/exp OR dialysis OR hemodialysis OR haemodialysis OR 'peritoneal dialysis' OR 'renal replacement therapy' AND ('erythropoiesis stimulating agents' OR 'recombinant human erythropoietin' OR rhuepo OR darbepoetin OR epoetin OR erythropoietin OR 'recombinant erythropoietin'/exp OR 'novel erythropoiesis stimulating protein'/exp)) AND 'randomized controlled trial'/de
